# Cholinium amino acid-based ionic liquids

**DOI:** 10.1007/s12551-021-00782-0

**Published:** 2021-01-14

**Authors:** Andrea Le Donne, Enrico Bodo

**Affiliations:** Chemistry Department, University of Rome “La Sapienza”, Piazzale A. Moro 5, 00185 Rome, Italy

**Keywords:** Ionic liquids, Biocompatible ionic liquids, Amino acids, Cholinium

## Abstract

Boosted by the simplicity of their synthesis and low toxicity, cholinium and amino acid-based ionic liquids have attracted the attention of researchers in many different fields ranging from computational chemistry to electrochemistry and medicine. Among the uncountable IL variations, these substances occupy a space on their own due to their exceptional biocompatibility that stems from being entirely made by metabolic molecular components. These substances have undergone a rather intensive research activity because of the possibility of using them as greener replacements for traditional ionic liquids. We present here a short review in the attempt to provide a compendium of the state-of-the-art scientific research about this special class of ionic liquids based on the combination of amino acid anions and cholinium cations.

## Introduction

Amino acid (AA)-based ionic liquids (AAILs) have emerged recently as a versatile and peculiar class of ionic liquids which have attracted attention from different fields of research owing to their biocompatibility, to the relative low cost of their synthesis, and to the availability of the reagents (Fukumoto and Ohno 2006; Ohno and Fukumoto 2007; Kirchhecker and Esposito 2016; Herrera et al. 2018; Ruivo et al. 2018). Ionic liquids (ILs) are nowadays widely known as a very varied and multifaceted set of different materials whose potential applications range from electrochemistry to medicine and from industrial processes to biomass processing agents. It is not easy to tell precisely what properties define an IL. In the recent past, ILs have been loosely defined as salts with low melting or glass transition points which display all of the following functional physico-chemical properties: solvation capabilities, ionic conductivity, low vapor pressure, and high thermal stability (Hayes et al. 2015). It is also true that, due to the sheer number of available molecular ion combinations that give rise to an IL, many of them show only a subset of these generic properties. For a more in-depth discussion of which properties actually define an ionic liquid, see the discussion by MacFarlane and Seddon (2007). Since their inception in the research field (Chum et al. 1975), ILs have been sometimes considered inherently “green” solvents, while recent developments have shown that their biodegradability can be low and their toxicity very high, especially for the ones which are based on fluorine compounds (Scammells et al. 2005; Petkovic et al. 2011).

Once realized that ILs are less environment-friendly than expected, part of the IL research has steered toward the quest for green and truly biocompatible ILs. One of the earliest examples of a fully biocompatible ionic compound can be traced back in 2004 (Abbott et al. 2004) where the coupling of choline chloride with organic, naturally occurring substances such as succinic and oxalic acids has been shown to produce a completely biocompatible ionic salt with melting points below 100 °C. Since then, it has become clear that a cholinium cation (Ch) might represent a competitive alternative to imidazolium both in terms of costs and in being a greener component in terms of biodegradability (Morandeira et al. 2017). At the same time, it was realized that naturally occurring organic acids could be used to replace the not environmentally benign, fluorinated anions such as Tf_2_N of SF_6_ (Fukaya et al. 2007). Other ways to improve the green character of ILs lie in the use of renewable and available compounds for their synthesis such as naturally occurring amino acids (Hulsbosch et al. 2016). These bio-based ILs would ultimately be suitable to fully realize a green chemistry cycle starting from their synthesis and ending to their use as reaction agents (Plaquevent et al. 2008; Gomes et al. 2019).

In cholinium-amino acid ionic liquids (ChAAILs), the deprotonated form of an amino acid is coupled to the cholinium cation. Given that, formally, the ensuing IL can be thought as emerging from the reaction of a base (choline hydroxide) and the amino acid, ChAAILs are part of the subclass of ILs known as protic ionic liquids (PILs), i.e., those substances arising from an acid base reaction or proton transfer. One of the key features of PILs is the presence of protons that spawn a hydrogen bonding network and can, at least in principle, act as efficient charge carriers. The possible chemical activity of protons in PILs has important effects not only on their nanoscopic structure (Bodo et al. 2014; Low et al. 2019) but on the bulk properties as well (Kreuer 1996); hence, it has spawned and intense research for the use of PILs as conductive materials in electrochemistry (Khan et al. 2017; Kasprzak et al. 2018; Shmukler et al. 2018; Liu and Yu 2019). It is worth noting that, among the very first attempts to obtain a fully biocompatible ILs, a different approach had also been implemented where the AA is protonated and acts as the cation (Tao et al. 2005, 2006).

In this short review, we will survey almost exclusively the class of liquids based on AA anions and the cholinium cation. In these liquids, biocompatibility stems directly (or sometimes is postulated) from the metabolic nature of the composing molecular ions. AA is the building unit of proteins and an essential component of our food. Choline (once known as vitamin B4) is a metabolite directly synthesized by the human body and is also an essential dietary nutrient. In particular, due to their reduced cost and greener properties, ILs based on cholinium have gained attention as solvent in the field of organic synthesis (Gadilohar and Shankarling 2017), and, recently, a short review focusing on their structural properties has been published (Gontrani 2018).

## Synthesis and physico-chemical properties

The first synthesis of ChAAILs was reported by Ohno et al. (2007), by Moriel et al. (2010), and by Liu et al. (2012b). The most common synthetic route is based on a simple neutralization reaction between a water solution of [Ch][OH] and the amino acid. The IL is obtained as a result of the acid-base reaction and purified by further water removal. A more controlled synthetic route employing a titration has been successively reported (De Santis et al. 2015). After it was noted that the [Ch][OH] solution is corrosive, reactive, and more expensive than [Ch][Cl] and that the titration procedure used by De Santis et al. (2015) is time-consuming, another synthetic variant has been recently proposed (Zhang et al. 2019; Latini et al. 2019) where they can be obtained via an ionic metathesis between potassium AA salts and [Ch][Cl] in ethanol. A key efficiency factor in this new synthetic route is that it employs the cheap [Ch][Cl] reagent and has a high efficiency, which makes this synthesis appealing for large-scale production or industrial applications.

An excerpt of the physical data that are available on these liquids is shown in Table 1 where we have picked a few examples for representative ChAAILs. The densities of these liquids have been found to lie within a limited range between 1.1 and 1.2 g cm^−3^. Densities do not simply correlate with molecular weights because the presence of multiple hydrogen bonding features is a crucial factor in determining the cohesive state of these substances.

**Table 1:** Excerpts from published data of some physical properties of ChAAILs

AA	Density (g cm^−3^)		Conductivity (μs cm^−1^)		Viscosity (mPa s)			*T*_g_ (°C)	*T*_d_ (°C)
Gly	1.14^a^	1.15^b^	67.7^a^	90.6^b^	182.3^a^	1230^b^	121^c^	− 61^c^	150^c^
Ala	1.11^a^	1.13^b^	21.3^a^	74.1^b^	385.6^a^	720^b^	163^c^	− 56^c^	159^c^
Pro	1.12^a^	1.14^b^	0.3^a^	7.5^b^	10,643.8^a^	9810^b^	500^c^	− 44^c^	163^c^
Ser	1.19^a^	1.20^b^	9.3^a^	17.5^b^	11,543.7^a^	12,500^b^	402^c^	− 55^c^	182^c^

^a^From (Tao et al. 2013)

^b^From (De Santis et al. 2015)

^c^From (Liu et al. 2012b)

Glass transition temperatures have been reported by Liu et al. (2012b) and lie in a wide range from − 74 to − 10 °C, depending on the anion structure. In contrast, De Santis et al. (2015) found [Ch][Asp] and [Ch][Glu] to become liquid only at 90 °C, while they were glassy solids at 25 °C. Decomposition temperatures have also been measured (Liu et al. 2012b) and turned out to be much higher than 100 °C for all compounds, hence comparable to traditional ILs. The high decomposition temperature is an indicator of the high ionicity degree within the liquid and of how the hydrogen bonding network can be effective in stabilizing the molecular constituents.

The measurements of viscosities show significant discrepancies between the published data. In the work by Liu et al. (2012b), all viscosities were found to lie within 121–5640 mPa·s at 25 °C, but much higher values were found by subsequent determinations (see Table 1, for an example). Despite differences in the actual data, it is clear that an increase in the size of the anion increases viscosity, probably because of stronger van der Waals interactions. In addition, the existence of a protic side chain (Cys, Asp, etc.) seems to be able to induce additional hydrogen bonding activity that greatly enhances viscosities. A recent study by Moosavi et al. (2019) using molecular dynamics provides calculated viscosities that are compatible with those from Liu et al. (2012b).

Conductivities are inversely related to viscosities and, despite numerical differences, have been found to loosely correlate with the anion size and its ability to spawn hydrogen bonding, two effects that tend to decrease the conductive performance.

Despite discrepancies in the measured data (that could be explained by different amounts of water contamination or by the typical metastability of some of the physical states of the compounds), a very clear picture emerges: ChAAILs are a series of homologous compounds whose molecular components feature only minor differences but present huge differences in the resulting bulk properties. Finding a simple correlation between the molecular characteristics and the bulk properties is not easy, and the latter seem to stem from a set of subtle molecular effects which are not simple to evaluate: many body effects, charge transfer phenomena, hydrogen bonding, and tautomerization reactions (see Le Donne et al. (2018) for a more in depth discussion).

## Toxicity and biodegradability

The toxicity of ionic liquids and their activity toward living organisms are a very complex subject (Wang et al. 2007; Zhao et al. 2007; Costa et al. 2017; Kumari et al. 2020), and it would be unpractical to list here a comprehensive list of references. The nature of the interactions that drives the action of ILs on living cells has been recently summarized by Kumari et al. (2020). Many traditional ionic liquids have been revealed to be toxic for living species (Bernot et al. 2005; Docherty and Kulpa Jr. 2005; Latała et al. 2005; Peric et al. 2013). This inherent toxicity of many ILs presents a twofold issue where, from a biotechnological standpoint, the high activity of ILs toward microorganisms hinders their use as pharmaceuticals, but their toxicity opens new vistas for the implementation of ILs as antibacterial and perhaps antifungal agents (Ibsen et al. 2018; Zandu et al. 2020).

The toxicity and biodegradability of several ChAAILs have been reported since their first appearance in the field by Hou et al. (2013b), and both were found to be better than traditional ILs. A subsequent analysis by Foulet et al. (2016) showed that ChAAILs can be classified as non-toxic (or even practically harmless for [Ch][Pro]) and, as expected, that their anti-microbial activity is very low. Another study based on the antimicrobial activity of a set of several ChAAILs demonstrated that those with AA anions of high molecular weight (Glu, Arg, Phe, Try) are practically harmless to various bacterial cultures (Yazdani et al. 2016).

The advantages of using ILs in the pharmaceutical industry are associated with their use as solvents to solubilize poorly soluble drugs, thus improving delivery, and with their potential in reducing typical problems affecting solid-state drugs such as polymorphism and bioavailability (Md Moshikur et al. 2020). The use of biocompatible ILs such as ChAAILs, in this context, has obvious advantages because they allow one to circumvent the toxicity issue of other ILs. For this reason, their use as a potential drug carrier has been explored, and they were found to improve the solubility of active pharmaceutical principles (APs) and to increase their capability to penetrate the cell membranes. In particular, [Cho][Phe] and [Cho][Glu] have been tested as excipients to enhance loading and solubility of poorly soluble APs (Caparica et al. 2018), while they did not increase the cytotoxicity of the preparations. Very recently, it was found that [Ch][Gly] and [Ch][Ala], even in small amounts, can significantly increase the solubility of ibuprofen without an increase in cytotoxicity (Yuan et al. 2020). Another recent study aimed at investigating the applicability of ChAAILs as excipients for the delivery of rutin as a potential anticancer agent (Caparica et al. 2020), and it was found that these ILs can act as safe functional excipients that are non-toxic and act to enhance the AP solubility while preserving its pharmacological activity.

## Interaction with complex biological molecules

ILs consisting of biocompatible cations and anions are of great interest for biotechnology and protein chemistry because they can serve as solvents potentially able to improve protein solubility, crystallization, and stability and to ease extraction and separation (Patel et al. 2014; Wakayama et al. 2019). Out of their native environments, proteins are often unstable. During an extraction, due to the change of environment, the extracted proteins may not function properly. It therefore follows that, for practical applications, the possibility of maintaining protein integrity for a long time outside their native environment is crucial. In other words, the nature of the “storage” environment is the key to preserve proteins and their bioactivity. Unfortunately, protein-IL interactions are very complex, and, at the moment, it appears that a generalization of the observations outside particular systems is not yet possible (Benedetto 2017).

Guncheva et al. (2019) have explored the stabilization of the monomeric form of insulin in ChAAILs (Asp, Glu, Lys, Arg) and have found that, in accord with previous experiments with other ILs based on imidazolium, protein aggregation is prevented, although a partial denaturation at the expenses of α-helices was observed.

It is generally assumed that ChAAILs act as gentle solvating agents for biomolecules. In support of this claim, a computational study (Kumari and Kashyap 2019) shows that the presence of the simplest form of ChAAIL, cholinium glycinate ([Ch][Gly]), does not disturb the structure and stability of lipid bilayers as much as traditional ILs based on imidazolium cations. Also, studies on the solvation of DNA (Sahoo et al. 2018) have shown only a minor binding affinity (~ 4 kcal/mol) of the ChAAIL for the macromolecule, whereby the Ch cations and not the amino acids were the main coordinating agents. Contrasting results have however been obtained by Bisht et al. (2018) where the stability of stem bromelain has been studied in the presence of both [Ch][Br] and [Ch][Gly]: unexpectedly, [Ch][Gly] proved to be a poor stabilizer for the enzyme when compared to [Ch][Br]. More recently Kumar et al. (2020) have shown that the replacement of the tetraalkylammonium cations with cholinium in AAILs can provide a better stabilizing environment for serum albumins.

The traditional techniques for protein extraction such electrophoresis, ion exchange, and chromatography are expensive, complex, and difficult to implement on large scales. Liquid-liquid separation systems may offer a cheap substitute. For example, aqueous two-phase systems, where polymer/IL or salt/IL mixtures are dissolved in water above critical concentration, can provide a cost-effective and environmentally friendly alternative (He et al. 2012; Benedetto and Ballone 2016; Zeng et al. 2016). Since biocompatibility and low toxicity are crucial in these applications, several research efforts have been directed toward ChAAILs as alternative solvents (Song et al. 2015, 2017; Wang et al. 2016a; Zafarani-Moattar et al. 2019). Aqueous biphasic systems consisting of ChAAILs together with a water solution of polypropylene glycol seem to be a nearly optimal combination because of the ability to preserve the protein enzymatic activity and because of the general safety of the additives that could contaminate the extracted product. ChAAILs based on the use of anionic dipeptides such as [Ch][Phe-Phe] has shown superior performances in the extraction and recovery of metabolites from microalgae processing (Morandeira et al. 2020). The peculiar biocompatibility of ChAAILs has been also exploited to construct especially designed biosensors based on Gly, Ser, Phe, and His which showed good reliability, sensitivity, limit of detection, and linearity of range (Zappi et al. 2019).

With the persistent reduction of petroleum-based resources, there has been a worldwide increase in the search for alternative energy sources. In this context, biomasses represent a particularly attractive source of energy because of their availability, but their use is hampered by the difficulties and by the processing costs of extremely recalcitrant materials such as lignin and cellulose and the energy needed for it (Carlson et al. 2008). It is well-known that ILs can efficiently dissolve a large number of macromolecules otherwise difficult to solubilize (see the paper by Sun et al. (2009) and references therein) and that the extensive hydrogen bonding network that characterizes PILs could been exploited to dissolve compounds which normally require very harsh conditions and highly toxic solvents.

The ability of dissolve lignin, xylan, and cellulose was investigated for ChAAILs by Liu et al. (2012b). The lignin dissolution reached satisfactory values for most of the ChAAIL at 90 °C (the solubility is more than 140 mg g^−1^), but the dissolution of xylan and cellulose was much poorer with solubility values lower than 5 mg g^−1^.

Hou et al. (2013a) have reported that lignin extraction from sugar cane reached 44% using a mild condition treatment based on increasing IL concentrations of a water solution of [Ch][Lys]. Wang et al. (2016b) used a water solution of [Ch][Leu] to extract flavonoids and pectin from ponkan peels at room temperature. In a recent paper, To et al. (2018) have shown that under very mild conditions (70 °C), a water solution of a set of chosen ChAAILs (Arg, Gly, Lys, and Phe) is able to extract 50% of lipids from algae biomasses. Among the AA anions, the most efficient was argininate, which yielded the best extraction performances. The structure of the argininate anion with its 6 hydrogen-bonding sites is very likely the responsible for its effectiveness.

Scarpellini et al. (2016) presented an exploratory study for paper-based cultural heritage conservation through the use of ChAAILs. They showed that a treatment with [Ch][Gly] increases the tensile strength of the fiber due to the action of the IL when the paper is subjected to artificially accelerated aging.

## Use as lubricants

The first work on a tribological use of traditional ILs dates back to 2001, with a study on two different imidazolium tetrafluoroborates ILs, which showed excellent friction reduction, high load-carrying capacity, and anti-wear performance (Ye et al. 2001). The main mechanisms beyond these excellent tribological properties have to be traced in the high adhesion energies of IL onto the surfaces that induce the formation of layers between them. The possible use of ILs as lubricants focused on ILs containing halogens and specifically those made using [BF_4_]^−^ and [PF_6_]^−^ anions, but these compounds can undergo hydrolysis by moisture and generate corrosive and toxic hydrogen fluoride. The ChAAILs emerged only recently as a greener type of lubricants. The combination of two ChAAILs with lignin (Mu et al. 2015) has been investigated to evaluate the tribological properties of these mixed systems. The use of lignin as an additive improves the thermal stability and the anti-corrosive properties of the resulting mixture, thus providing excellent performance when used with commercial aluminum and iron boards.

The tribological properties of five ChAAILs have been explored using steel/steel and copper/steel contacts and turned out to be comparable to conventional IL such as [C_6_mim][NTf_2_] (Jiang et al. 2018). The lubricant action has been confirmed as due to the formation of a physically adsorbed thin film of liquid on the metal surface.

In the framework on finding non-toxic lubricants, Zhang et al. (2018) have explored, in addition to the tribological properties, the toxicity of several ChAAILs against three aquatic organisms: brine shrimp, zebrafish, and green algae. The results show that their toxicities are significantly lower than that of traditional ILs such as 1-butyl-3-methyl imidazolium tetrafluoroborate and that ChAAILs can be used as high-performance and environmentally friendly lubricants. Hua et al. (2020) have shown that the frictional properties of [Cho][Pro] can be controlled by altering the environmental humidity. The viscosity of the ChAAIL increases at higher humidity, while it decreases when water is evaporated.

## ChAAIL as candidates for CO_2_ absorption

The worldwide issue of CO_2_ emissions linked to anthropogenic global warming is one of the major concerns of this century (Stern 2007). Technologies that can offer sustainable solutions have undergone intense research, and ILs do play a role as materials which is worth investigating as a medium for CO_2_ absorption and storage (MacDowell et al. 2010). ILs can be incorporated into existing processing technologies, especially coal-fired power stations, to aid the separation of CO_2_ from flue gas (Gurkan et al. 2010a; Smiglak et al. 2014), and both theoretical and experimental studies have been performed on how ILs can interact and react with CO_2_ molecules. In general, the use of ILs as CO_2_ capture agents presents two drawbacks: limited maximum gravimetric absorption (7–9%) and slow absorption kinetics due to the high viscosity (Bates et al. 2002), although results have been reported with a gravimetric capacity of more than 16% (Wang et al. 2010).

The challenge lies with fine-tuning IL specific reactivity and selectivity for CO_2_ trapping while, at the same time, providing a recyclable and biocompatible material. This is essential in order for the technology to be economically feasible and for scaling to larger and greener applications. An issue in the chemisorption of CO_2_ is the overall economic viability of the process because it is not a profitable industry (MacDowell et al. 2010). Even with incentives such as carbon credit schemes, it seems not plausible to eliminate the costs of carbon capture, and therefore any new IL proposed for CO_2_ absorption purposes must be inexpensive.

Two different CO_2_ absorption processes in ILs have been reported in the literature (Mumford et al. 2017; Song et al. 2019): ion structures that can react and combine with CO_2_ (chemisorption) or compounds that can physically dissolve CO_2_ (physisorption). Our interest for the present review lies in the former where CO_2_ is removed from flue gas using a chemical reaction that transforms it into a component of the liquid. The traditional technologies used to capture CO_2_ in industry settings consists of a chemical reaction of the gas with a solution of ethanolamine, which has a low cost, and a gravimetric capacity of about 7%, but has major problems related to toxicity and corrosion (Rao and Rubin 2002).

A possible alternative to the ethanolamine-based approach is the use of amine-functionalized ILs which react with CO_2_ molecules with the formation of carbamate species and producing improved reaction stoichiometries (Camper et al. 2008; Vijayraghavan et al. 2013; Li et al. 2019). This method tends to cause large changes in solvent viscosity, yet this can be circumvented via counterions that do not promote the formation of H-bonded networks or by water addition.

Within AAILs (also those with cations different from [Ch]^+^), absorption can occur to various extents depending on the physical conditions and on the IL molecular composition: absorption ranges from 0.5 mol of CO_2_ per mol of IL (2:1 mechanism) up to 1 mol of CO_2_ per 1 mol of IL (1:1 mechanism) and sometimes higher molar fractions (1:2 mechanism). It is clear that this range of results implies the existence of different reaction mechanisms.

**Scheme 1:**
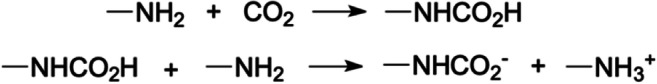
General reaction for the addition of CO_2_ to an amino group. The first step leads to the formation of a carbamic acid derivative. The second leads to a carbamate

CO_2_ capture in these liquids is achieved by exploiting the –NH_2_ group on the AA anions. The general reaction of amines with CO_2_ is known (Goodrich et al. 2011; Firaha and Kirchner 2016; Li et al. 2018) and can be summarized as in Scheme 1.

Whether the reaction proceeds with a 1:1 or 2:1 stoichiometry depends on what extent the second reaction takes place after the initial carbamic acid formation. In order to enhance absorption, it is necessary to favor the carbamic acid formation instead of carbamates. Gurkan et al. (2010b) explored two AAILs based on Pro and Met which have been coupled with a phosphonium cation. The absorbed molar ratio of CO_2_ per mol of IL was 0.9, thus increasing by a factor of 2 the efficiency of previous setups, thanks to the possibility of favoring a carbamic acid derivative instead of the carbamate one. The gravimetric capacity, though, was still low, about 6%.

Other authors have reported larger absorption capacity up to 1:2 M ratio. In a first work (Luo et al. 2019), a set AAILs with different basicity and steric hindrance was tested. It was found that the di-anion of a doubly deprotonated AA such as Asp showed a very high absorption capacity of 1.96 mol/mol IL at 30 °C and 1 atm. Along the same route, the activation of the carboxyl group in the AA using an electron withdrawing group has also been tested increasing load factors up to 1.69 mol/mol (Chen et al. 2016). Ammonium-based cations with longer side chains instead of cholinium have also been tested as a viable route to achieve 1:2 absorption ratios (Saravanamurugan et al. 2014). These works all incorporate AA anions in the IL, but the nature of the cationic partner can raise an environmental issue if it is toxic to the ecosystems. For this reason, ChAAILs have attracted the attention of researchers in this field because of their biodegradability and inexpensive synthetic procedure due to the relative abundance of their components (Gurkan et al. 2010b; Liu et al. 2012a).

**Scheme 2:**
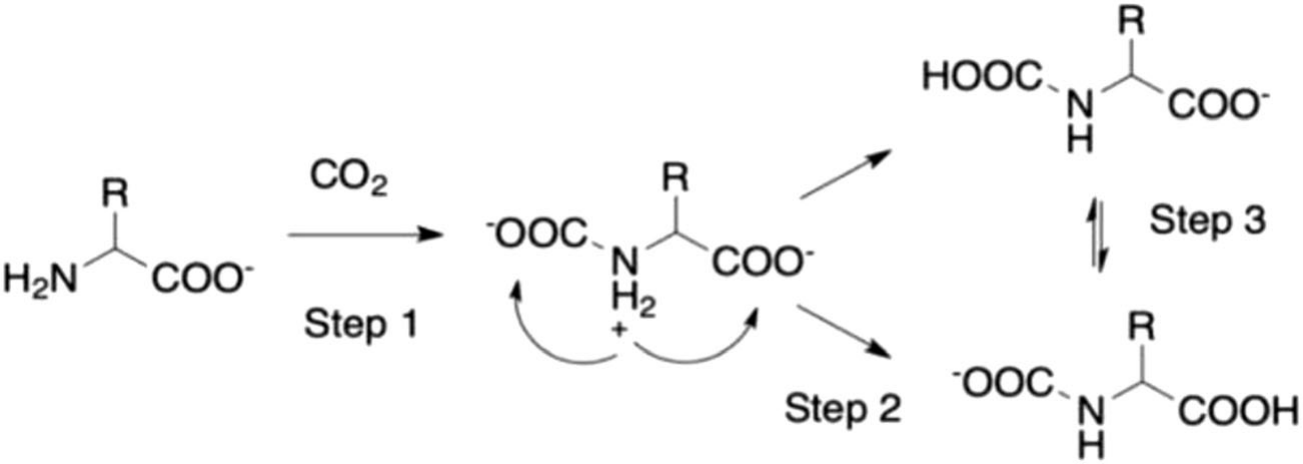
General reaction mechanisms for the addition of CO_2_ to an AA anion. The first step leads to the formation of a zwitterionic adduct that evolves via proton transfer to a carbamic/carbamate derivative which, in principle, are in equilibrium due to tautomerization reactions

The general mechanism for addition of a single molecule of CO_2_ to an AA anion is reported in Scheme 2.

This reaction can be divided into two steps as shown in Scheme 2: (i) a pre-reaction complex formation (which has zwitterionic character) and (ii) a proton transfer (PT) to either carboxylate terminals. In principle the two tautomeric forms of the resulting product are in equilibrium (3), in practice, one form is often markedly more stable than the other, and the final product is only one thereby favoring either a 1:1 or 2:1 stoichiometry in the final absorption. Several approaches based on computational modeling have been used to unravel the detailed mechanism of the reaction above (Sheridan et al. 2018).

**Scheme 3:**
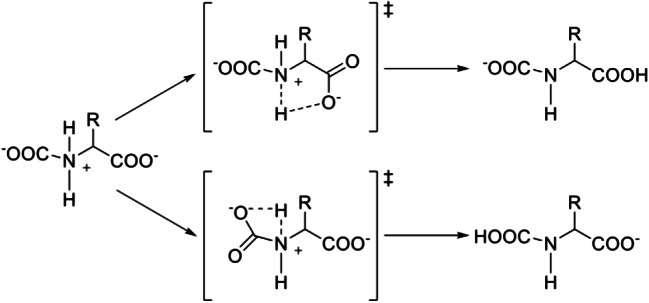
Possible mechanisms of the proton transfer step with a 5-member ring or a 4-member ring in the transition state

Shaikh et al. (2016, 2020) showed the existence of barriers associated with the proton transfer step that leads the pre-reaction zwitterion to the final carbamate. Mercy et al. (2016) and Onofri et al. (2020) have found that the proton transfer step in [Ch][AA] can occur via two possible reaction pathways that differ by the size of the ring that is formed in the transition state as shown in Scheme 3. The mechanism based on a 4-member ring is highly disfavored due to very high barriers, while the one with a 5-member ring has a low activation energy of only few kcal/mol.

The main problem is that the existing ChAAILs are highly viscous, limiting their applicability as agents incorporating CO_2_ because of its limited diffusion in the liquid. Attempts have been made to reduce the viscosity either reducing the strength of the hydrogen bonding network (Luo et al. 2016) or using ether substituents (Goodrich et al. 2011). In the latter case, the [Ch][Lys] compound has been shown to maintain a high capture capacity of 1.62 mol/mol. The use of the doubly deprotonated forms of certain AA anions has also been explored as a tool to reduce or control the overall viscosity of the final mixture (Pan et al. 2018). Attempts at using co-solvents have been made in order to reduce the viscosities of ChAAILs. Davarpanah et al. (2020) have attempted dissolution of the IL into another solvent with high boiling point such as DMSO, but the dilution of the absorbent material led to a reduced absorption capacity (with 12.5 wt% IL in DMSO, only 0.3 mol CO_2_/mol IL of CO_2_).

**Scheme 4:**
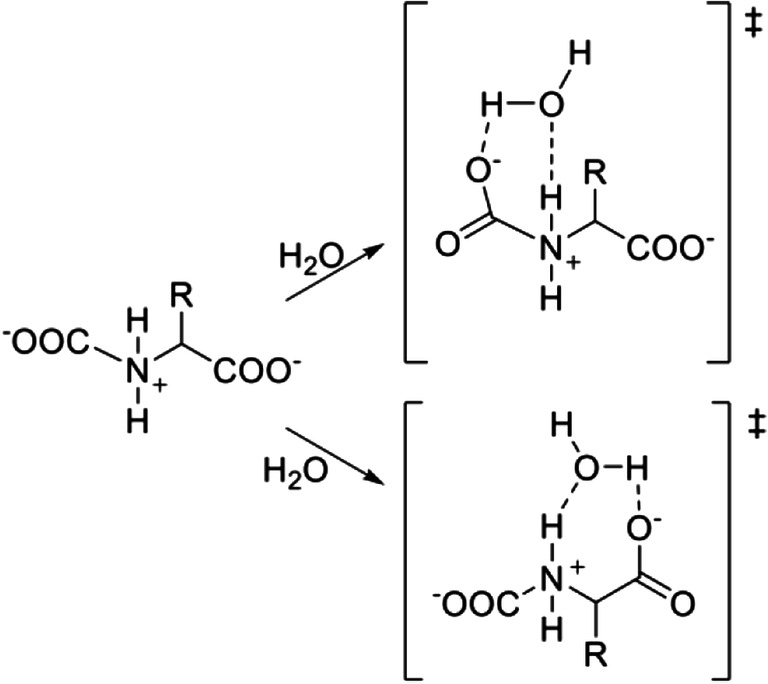
Possible mechanisms of the proton transfer step with a 6-member ring or a 7-member ring in the transition state when including a water molecule

The addition of water to ILs (Li et al. 2018), in the context of CO_2_ absorption, is known to have beneficial effects in reducing the viscosity of the absorbent fluid. Water seems to play a two-faced role since it works by reducing viscosity and hydrogen bonding, thus increasing the diffusion of CO_2_, as well as removing phenomena that render the reaction site less available, but it may also act as a catalyst for the overall CO_2_ absorption reaction by assisting a more efficient PT. When a single water molecule was included in the transition state computational evaluation, it effectively lowered the kinetic barriers of this step (Li et al. 2018; Shaikh et al. 2020; Onofri et al. 2020) by reducing the strain on the transition state cycle as shown in Scheme 4.

Using a completely different chemical process, a set of ChAAILs has been examined by Saptal and Bhanage (2017) where 9 AA anions were used as solvent catalysts in order to help the chemical fixation of CO_2_ into cyclic carbonates. The binary system based on [Ch][AA]/TBAI, in particular, generated deep eutectic solvents (DESs), which were found to be highly active at atmospheric pressure with the hydroxyl functional group activating the epoxide ring and the amino group activating the CO_2_ molecules for reaction. In these setups, the catalyst and the co-catalyst are both recyclable up to five times without loss of catalytic activity.

## Structure and computational studies

**Fig. 1:**
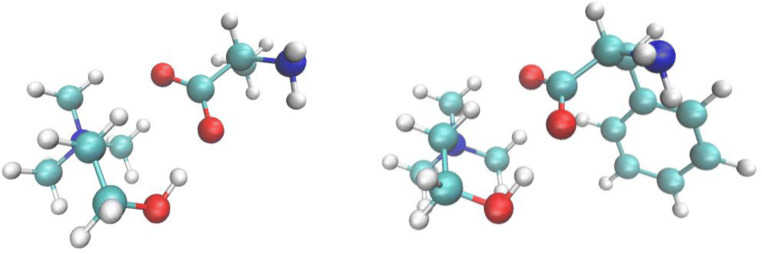
Example structures for the [Ch][Ala] (left) and [Ch][Phe] (right) ionic couples. Their interaction energies as reported by Benedetto et al. (2014) are 106 and 103 kcal/mol, respectively

The many applications of ChAAILs have attracted the attention of computational chemists. Following the early synthesis and physical characterizations, Benedetto et al. (2014) have implemented a DFT-based study of 8 different AA anions focusing on the stability of the isolated ionic couples. Along the series of 8 AA anions, the average [Ch]^+^[AA]^−^ interaction energy shows only minor variations and turns out to be very large, around 100 kcal/mol. The anion-cation geometric binding motif in the isolated ionic couples also is rather invariant along the different AA anions and is reported in Fig. 1 for two selected examples. Apart from the obvious electrostatic interaction, the binding interaction is provided by a hydrogen bond between the cholinium hydroxyl and the AA carboxylate.

The same kind of approach was also used by del Olmo et al. (2016) extending the series of AA anions to 20 and by calculating, via the COSMO approach, bulk properties such as density and viscosity. While the data of the densities were in line with experiments, the viscosities did not match the experimental measurements, although the latter (Liu et al. 2012b; Tao et al. 2013; De Santis et al. 2015) showed noticeable differences among them. Recently, Moosavi et al. (2019) have performed molecular dynamics (MD) simulations on the ChAAILs containing [Ala], [β-Ala], and [Phe] and computed the viscosities via the autocorrelation of the stress tensor: their data are compatible with the ones originally reported by Liu et al. (2012b), but it is well known that modeling the frictional properties of ILs using fixed charges schemes has certain accuracy limits due to the neglect of polarization and charge transfer effects.

The original conclusion that the main binding motifs in these liquids stem from the cation/anion coordination through strong hydrogen bonding has been further confirmed by using MD simulations and X-ray scattering data in a paper by Russina et al. (2016) where the subtle effects due to multiple coordination patters have also been described. The bidentate nature of the carboxyl unit on the AA anion can induce the existence of two kinds of coordination patterns: in the first, one anion coordinates one cation interacting with the hydroxyl and the quaternary ammonium. In the second, a single anion can coordinate simultaneously two cholinium cations by hydrogen bonds.

From the earliest computational studies, it was clear that the ChAAILs structure was not as simple as initially thought. In particular, the emergence of low-Q peaks in the X-ray diffraction patterns seemed to indicate some kind of aggregation phenomena at the nanoscale which previous calculations on isolated dimers were not able to grasp (Campetella et al. 2015b, 2016b). The authors of these studies focused on Pro- and Phe-based ChAAILs where the presence of structured side chains could induce and enhance the formation of transient structures at the nanoscopic level. Interestingly, ChAAILs based on Phe anions showed low-Q peaks in their X-ray scattering pattern without having a long alkyl chain in their structure. The interpretation of the experimental diffraction data relied on the presence of correlations between second-neighbor groups (cation-cation and anion-anion) that seems to characterize the local structure in the fluids and that can induce the emergence of the spectral fingerprint of aggregation phenomena.

Analogous approaches based on a combined experimental and MD study by Gontrani et al. (2017) have elucidated, using different degrees of water dilution in the IL, the behavior of hydrogen bonding between opposite- and like-charge dimers and the intercalation structure of the final solutions. These findings agree with what was found by Shyama and Lakshmipathi (2020) where 8 [EMIM][AA] ILs were investigated by DFT calculations. Water tends to stick to anions, and only a high number of water molecules leads to the separation of the ionic dimers.

**Fig. 2:**
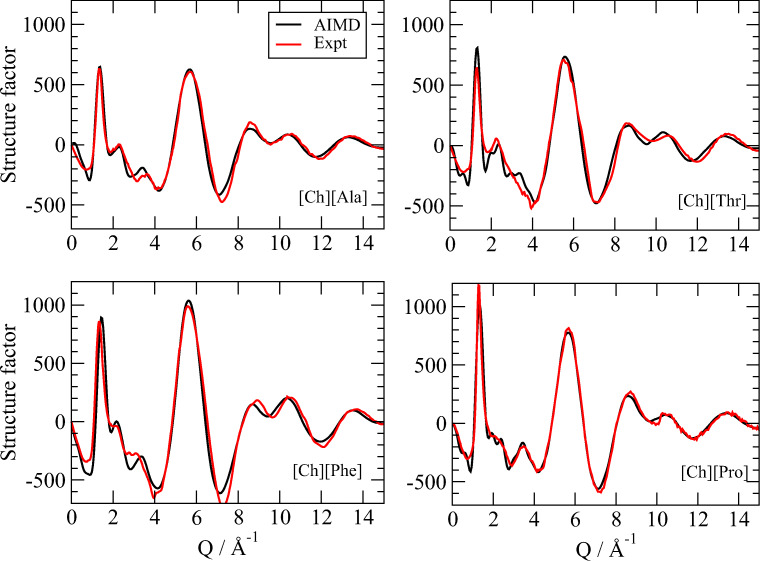
Total X-ray scattering structure factors as computed through AIMD compared to experimental measurements (no scaling involved) for [Ch][Ala], [Ch][Thr], [Ch][Phe], and [Ch][Pro]. See Campetella et al. (2016a) for more details

From the studies described above, it was clear that both the short- and long-range structure of these fluids was determined by a subtle balance between the weak interactions between either the molecular components with opposite charge or those with the same charge. Modeling such interactions requires necessarily a great accuracy in the calculation of the interatomic potentials because of the need to describe polarization phenomena and many-body effects. In order to properly include such effects, the MD studies must be based on a first principles evaluation of the electronic energy (ab-initio MD or AIMD). In these approaches, both the size of the systems and the time scales are much reduced with respect to traditional MD techniques, but the reliability of the outcomes is generally increased. AIMD has also the advantage of accounting for anharmonic motions and providing atomic charge fluctuations so that in addition to structural information such as X-ray diffraction patterns, IR absorption spectra can be calculated as well. Known IR absorption bands of functional groups are obviously sensible to the chemical surroundings, thus providing indications on the aggregation state (Tanzi et al. 2014). The structural and spectral properties of several ChAAILs have been explored by means of AIMD, by comparing the outcomes of the simulation with both X-ray (Campetella et al. 2016a) and with IR absorption and Raman measurements (Campetella et al. 2015a, 2018). An example of the structural information available and of their accuracy with respect to experimental data is reported in Fig. 2 where the ability of AIMD to almost perfectly reproduce the structure of the fluids should be apparent. Analogous comparison of computed data with experimental IR absorption profiles can be found in the works by Campetella et al. (2015a, 2018).

As suggested by a series of works by Ludwig and coworkers (Niemann et al. 2018, 2019, 2020; Khudozhitkov et al. 2019), the interactions between like-charge ions could be a crucial ingredient in those ILs that are characterized by a high degree of H-bonding interactions. Multiple and cooperative H-bonding features seems to be sufficient to overcome the natural Coulombic repulsion between like-charge ions, especially when the charge is delocalized over the molecular structure and the repulsion weakened by the surrounding dielectric response of the medium (polarization).

The possibility of having anionic aggregation and the extent to which like-charge interactions might contribute to the overall cohesive energy of few selected ChAAILs have been thoroughly explored in a series of papers by us and other authors. Even in the simplest AA anions with aliphatic side chains, the computational data do suggest the presence of conspicuous anion-anion pairing through H-bonding of their amino and carboxylate groups (Fedotova et al. 2019). Le Donne et al. (2018, 2019) using AIMD have found a rough but evident correlation between the association energies of the dimeric anion-anion structures and the measured viscosities of the corresponding ChAAILs. A recent work by Khorrami and Kowsari (2020) based on classical MD has further extended the study of these like-charge interactions to the mixtures of ChAAILs with water.

Until recently, the possibility of having anion-anion aggregation phenomena in ChAAILs was the result of computational evidence. Very recent, though, an experimental corroboration has been obtained from the fitting of the molecular structure of [Ch][Phe] to neutron diffraction data, thus confirming the theoretical hypothesis (Miao et al. 2020b). These experiments show that molecular segregation is present, as well as the existence of anion-rich domains which are controlled by inter-anion hydrogen bonds. These anionic aggregation phenomena might also be responsible for the low-Q correlations spotted in SAXS diffraction patterns which are otherwise difficult to attribute to more common effects such as apolar domain segregation in compounds which do not have long aliphatic chains (Miao et al. 2020a).

**Fig. 3:**
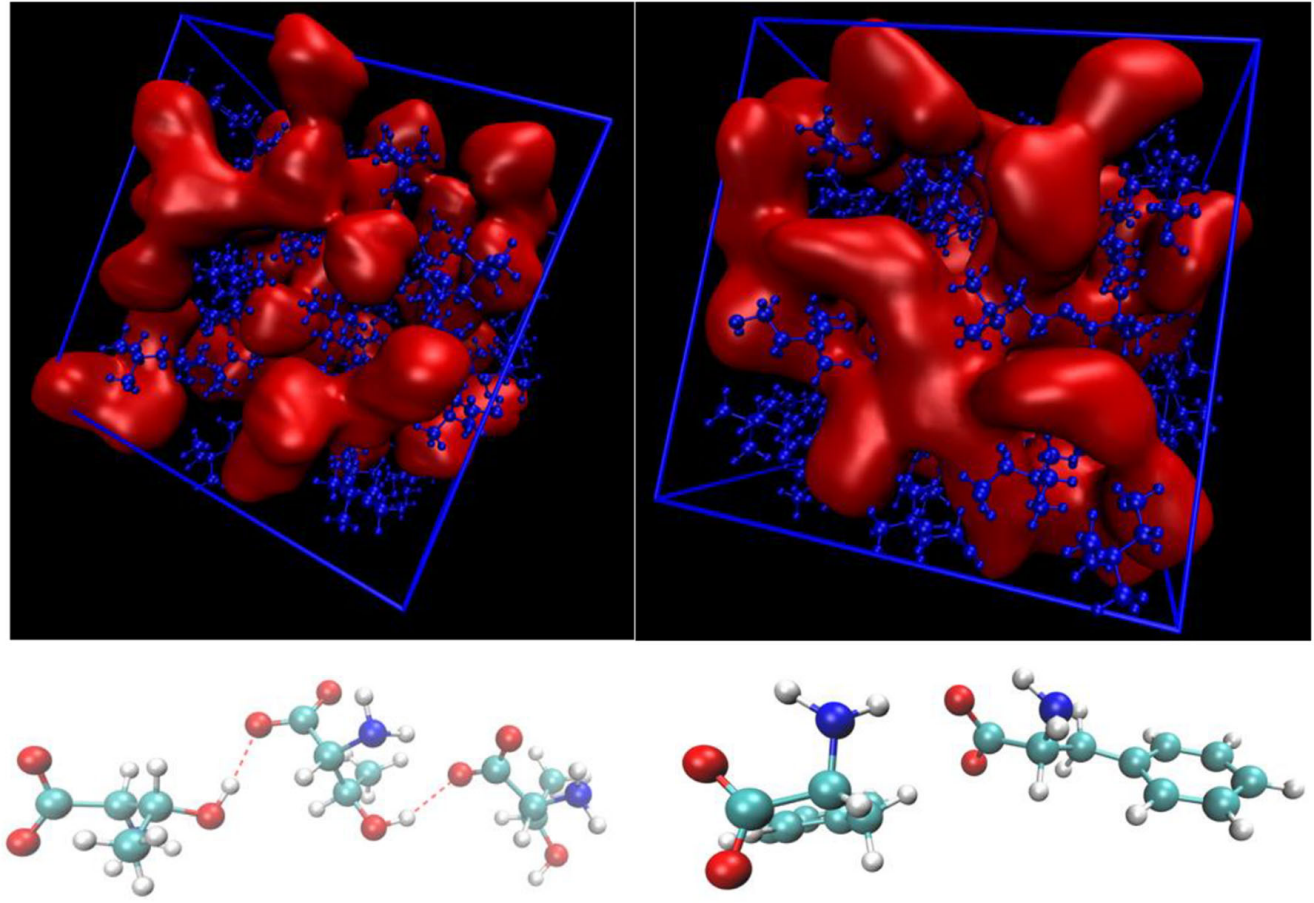
Top: snapshots of AIMD simulations containing [Ch][Thr] (left) and [Ch][Phe] (right). The anionic moieties are represented by the red iso-surfaces, while the cations correspond to the blue structures. The formation of oligomers of anions is evident as well as the nano-segregation between the oppositely charged moieties. Bottom: examples of hydrogen-bonded clusters of [Thr] and [Phe] anions as extracted from the simulations at the top. For details see Le Donne et al. (2019)

A pictorial example of these domains as they emerge from AIMD simulations is presented in Fig. 3 where the aggregation state of [Thr] and [Phe] anions is depicted using the atomic volumetric density in snapshots of the simulation. Examples of a trimeric [Thr_3_]^3−^ and of a dimeric [Phe_2_]^2−^ anionic structures are reported at the bottom.

**Scheme 5:**
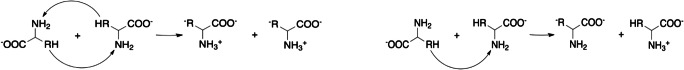
Possible tautomerization reactions in AA anions with protic side chains. On the left, we report the formation of two zwitterionic anions and on the right the formation of a dianion and a neutral species. Here R represents a protic group such as SH, COOH and PO_3_H_2_

Recent calculations by us (Campetella et al. 2017; Adenusi et al. 2020b) have shown that these anionic domains can host a rather complex chemistry due to proton transfer processes and to the respective tautomerization processes. In these works, it has been shown that AA anions containing an additional protic function, such as –SH, –PO_3_H_2_, or –COOH, can exist in the form of a zwitterionic-anionic tautomer where the additional proton has moved onto the –NH_2_ group. The presence of multiple partial charges on the same molecular ion further weakens the overall anion-anion electrostatic repulsion and allows for even tighter anion aggregation phenomena. The mechanism of two possible tautomerization pathways in the anionic moiety of AA anions with protic side chains is reported in Scheme 5.

PILs have been, since their inception in the landscape of ionic media, an important candidate for achieving highly conductive solvents especially tailored for electrochemistry (Lu et al. 2012). The problem with these substances is that their degree of ionicity and their ability to transport charge are very dependent upon the tendency of the involved molecular species to bind the proton. The correlation between quantities such as pK_a_ and proton affinities of the acid and base involved and the ensuing ionization degree and conductive properties of the fluid has been the subject of a great number of research activities, but it still eludes a simple generalization (Shmukler et al. 2020). The ChAAILs in which the AA anions have an additional protic function on the side chains and where the additional protons are mobile and give rise to the aforementioned tautomerization reactions might provide an exemplar ionized medium where fast charge transfer could be achieved by proton diffusion (Adenusi et al. 2020b) and point toward the development of novel conducting media (Adenusi et al. 2020a).

## Conclusions

ChAAILs are a class of biocompatible ionic liquids that have attracted in recent years a vast research effort because they are emerging as suitable candidate to supplant first-generation ILs in many technological compartments. In the present review, we have summarized the results of these research efforts. These compounds have been known to be a versatile replacement for traditional ILs in various applications ranging from their use as lubricants to being efficient solvents for biomolecules, from media for protein extraction and preservation to electrochemistry. In the last part of this work, we have also summarized the results of the studies dealing with their structure at a more fundamental level which resulted in understanding that their structure is far from being trivial and shows peculiar and interesting features such as segregation phenomena that might come from the aggregation of the anionic moiety due to cooperative hydrogen bonding effects. The most recent results show that the nanoscopic structure of the anionic component can be very different from the expected, hence pointing to interesting developments for the achievement of proton conductive highly ionized media.
